# Tear-Based Vibrational Spectroscopy Applied to Amyotrophic Lateral Sclerosis

**DOI:** 10.1021/acs.analchem.1c02546

**Published:** 2021-12-14

**Authors:** Diletta Ami, Alessandro Duse, Paolo Mereghetti, Federica Cozza, Francesca Ambrosio, Erika Ponzini, Rita Grandori, Christian Lunetta, Silvia Tavazzi, Fabio Pezzoli, Antonino Natalello

**Affiliations:** † Department of Biotechnology and Biosciences, University of Milano-Bicocca, Piazza della Scienza 2, 20126 Milano, Italy; ‡ Department of Materials Science, University of Milano-Bicocca, Via R. Cozzi 55, 20125 Milano, Italy; § COMiB Research Centre in Optics and Optometry, Via R. Cozzi 55, 20125 Milano, Italy; ∥ Bioinformatics Consultant, 15061 Arquata Scrivia, Italy; ⊥ NEuroMuscular Omnicentre (NEMO), Serena Onlus Foundation, Piazza Ospedale Maggiore 3, 20162 Milano, Italy; # NEMO Lab, Piazza Ospedale Maggiore 3, 20162 Milano, Italy

## Abstract

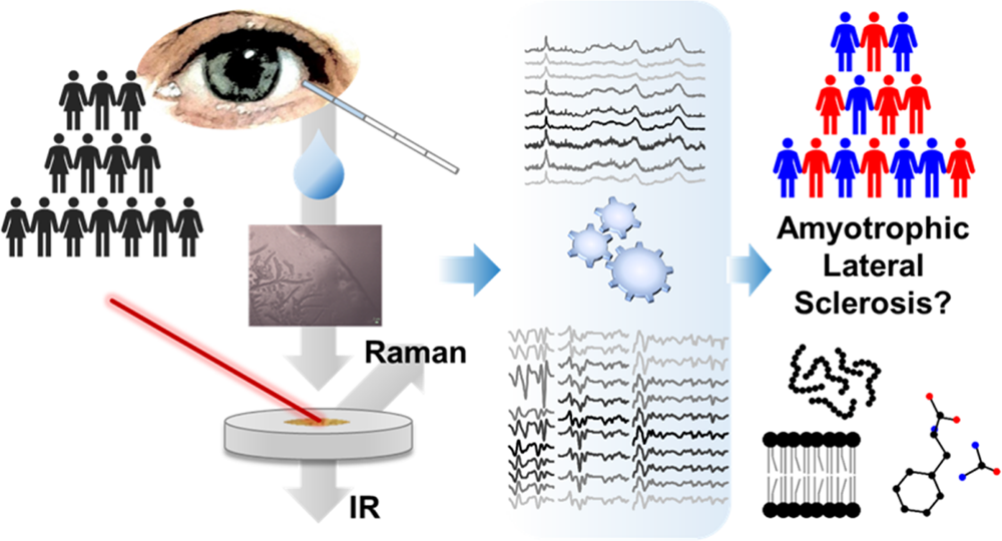

Biofluid analysis by optical spectroscopy techniques is attracting considerable interest due to its potential to revolutionize diagnostics and precision medicine, particularly for neurodegenerative diseases. However, the lack of effective biomarkers combined with the unaccomplished identification of convenient biofluids has drastically hampered optical advancements in clinical diagnosis and monitoring of neurodegenerative disorders. Here, we show that vibrational spectroscopy applied to human tears opens a new route, offering a non-invasive, label-free identification of a devastating disease such as amyotrophic lateral sclerosis (ALS). Our proposed approach has been validated using two widespread techniques, namely, Fourier transform infrared (FTIR) and Raman microspectroscopies. In conjunction with multivariate analysis, this vibrational approach made it possible to discriminate between tears from ALS patients and healthy controls (HCs) with high specificity (∼97% and ∼100% for FTIR and Raman spectroscopy, respectively) and sensitivity (∼88% and ∼100% for FTIR and Raman spectroscopy, respectively). Additionally, the investigation of tears allowed us to disclose ALS spectroscopic markers related to protein and lipid alterations, as well as to a reduction of the phenylalanine level, in comparison with HCs. Our findings show that vibrational spectroscopy is a new potential ALS diagnostic approach and indicate that tears are a reliable and non-invasive source of ALS biomarkers.

## Introduction

Amyotrophic lateral sclerosis (ALS) is one of the most severe neurodegenerative disorders characterized by degeneration of upper and lower motor neurons, leading to death in a median time of 3 years from onset. Most ALS cases are sporadic (∼90–95%), while the remaining 5–10% are familial, with the most common mutations affecting superoxide dismutase 1 (SOD1), transactive response DNA-binding protein 43 (TDP-43), RNA-binding protein FUS, and the hexanucleotide repeat expansions in C9ORF72.^1,2^ Non-motor pathways are also affected, and up to 50% of patients have detectable cognitive and behavioral changes, leading in about 15% of cases to a frank frontotemporal dementia.^3^

The diagnosis of ALS is achieved by the combination of clinical data and neurophysiological evidence of motor neuron degeneration, together with symptom progression, leading to a delay between onset and diagnosis that limits prompt intervention. Therefore, the discovery of new biomarkers easily accessible and quickly detectable represents a priority for early diagnosis, patient stratification, and evaluation of the therapeutic and rehabilitative effectiveness.^4^ Moreover, biomarkers that ensure a precise discrimination between diseased and healthy individuals can lead to radically new diagnostic tools and offer crucial insights into the pathogenic molecular mechanisms.^5^

Vibrational spectroscopies relying on Raman scattering and infrared absorption are label-free and non-invasive tools that already spur an enormous interest within biology and medicine alike. Interestingly, Fourier transform infrared (FTIR) and Raman spectroscopies can be applied not only to the characterization of isolated biomolecules but also of intact cells and tissues. When combined with multivariate analysis, these complementary vibrational techniques offer a powerful diagnostic tool for rapid “spectroscopic fingerprinting” of the sample offering a snapshot of the composition and structure of its main biomolecules. Then, the use of a microscope allows collecting the signal from a selected sample area with a spatial resolution that depends on the instrument characteristics. This property makes this approach particularly suitable for the investigation of samples characterized by a significant intrinsic heterogeneity. More recently, this approach has been extended to peripheral tissues and body fluids.^6−13^ The latter are gaining considerable momentum, particularly for future precision medicine and point-of-care diagnostics, due to low invasiveness and sustained availability.

In this context, tear fluid has been largely overlooked, notwithstanding its compelling advantages. Aside from being accessible, tears regenerate within a few minutes and are not contaminated by other biological fluids, for example, blood.^14^ Tear analysis for the diagnosis and monitoring of neurodegenerative disorders has emerged in recent years, particularly for Alzheimer’s and Parkinson’s diseases. However, further research is needed to explore the potential of this biofluid.^15,16^ Tear is a complex biofluid with more than 1500 proteins (with a total concentration of 6–11 μg/μL), 100 small metabolites, 150 lipid species, nucleic acids (including miRNAs), and electrolytes (Na^+^, K^+^, Ca^2+^, Mg^2+^, and Cl^–^ at concentrations ranging from 0.6 to 138 mM).^17,18^ Interestingly, also proteins known for their involvement in neurodegenerative diseases (including ALS) have been detected in tears.^18−21^

Motivated by these intriguing prospects and stimulated by recent suggestions regarding the ocular involvement in ALS,^22−25^ we turned our attention on vibrational spectroscopy of tears as a useful potential source of bioanalytes of this severe neurodegenerative disease.

Figure S1 shows a schematic of our blueprint. Tears are non-invasively collected from ALS-positive patients and healthy controls (HCs) and analyzed through FTIR and Raman spectroscopies. Classification was carried out by means of partial least-square discriminant analysis (PLS-DA), neural networks (NNet), and extreme gradient boosting (xgbTree). This process flow can identify the most significant spectral changes discriminating between ALS and HC tears. Specifically, we demonstrate a discrimination between the two sample classes with a strikingly high specificity (∼97 and ∼100% for FTIR and Raman spectroscopies, respectively) and sensitivity (∼88 and ∼100% for FTIR and Raman spectroscopies, respectively).

The assignment of spectroscopic markers of ALS to specific molecular classes can, in turn, provide new insights into this neurodegenerative disorder, including the role of protein misfolding and aggregation, as well as of metabolic alterations.

The feasibility of vibrational spectroscopy on tear fluid to screen for ALS emphasizes its prospects to eventually emerge as an effective diagnostic technique also for other neurodegenerative disorders and systemic diseases.

## Materials and Methods

### ALS Patients and Healthy Controls

All the ALS-diseased patients were clinically characterized (including the determination of the ALS Functional Rating Scale–revised (ALSFRS-r) total and domain scores) and genetically tested for the four main ALS mutations: SOD1, FUS, TARDBP, and C9ORF72, which were found in ∼9% of the enrolled patients (Table S1). Demographic and clinical features of HCs and ALS patients (Table S1), as well as inclusion and exclusion criteria, are reported in the Supporting Information.

### Tear Collection

Tears were collected by placing a glass capillary parallel to the lower meniscus. The typical volume which can be collected with this method is about 4–5 μL. This non-invasive method requires a few minutes, does not require extraction techniques, and allows repeated withdrawals from the same subject.

Collected tears were stored at −80 °C until use. Before spectroscopic measurement, tears were centrifuged at 7000*g* for a few seconds to collect the whole solution on the bottom of the test tube and to remove bubbles eventually formed in consideration of the low volumes employed.

### FTIR Microspectroscopy

For the micro-FTIR analyses, 2 μL of tears—from patients affected by ALS and from HCs—was deposed on a BaF_2_ window and dried at room temperature for about 30 min to eliminate the excess of water, which displays a very high absorption in the mid-IR.

Absorption spectra were obtained in transmission mode, between 4000 and 800 cm^–1^, by a Varian 610-IR infrared microscope coupled to a Varian 670-IR FTIR spectrometer (both from Varian Australia Pty Ltd) equipped with a mercury cadmium telluride nitrogen-cooled detector under accurate dry air purging. The variable microscope aperture was adjusted to ∼100 μm × 100 μm.

Measurements were performed at 2 cm^–1^ spectral resolution, 25 KHz scan speed, triangular apodization, and by the accumulation of 512 scan co-additions.

An overview of the FTIR spectra analysis is reported in Figure S2.

For each sample, different areas have been measured to explore the spectral heterogeneity (average absorption spectra are reported in Figure S2).

The average second derivative from 57 ALS-positive patients (962 spectra for the fern-like morphologies and 79 for the lipid granules) and from 36 HCs (521 spectra for the fern-like morphologies and 40 for the lipid granules spectra) were calculated (Table S1).

### Raman Microscopy

Raman measurements were carried out using a Horiba T6400 instrument in a single spectrometer configuration with an 1800 L/mm grating and a Si charge-coupled device cooled by liquid nitrogen. The laser excitation, operated at 532 nm wavelength and 6 mW power to avoid heat-induced degradation of the sample, was focused onto the specimen using a 100×/0.9 NA objective, which was also used to collect the Raman signal in the so-called backscattering geometry.

### Multivariate Analysis

Multivariate analysis was performed using R version 3.6.3. The FTIR second derivative spectra were processed by a fast algorithm for identifying multivariate outliers in high-dimensional data sets, as described in ref (26) and implemented in the R package *mvoutlier* version 2.0.5. Both FTIR data and Raman spectra have been tested for outliers. No outliers were found, so all spectra were retained for further analysis.

FTIR second-derivative spectra were split into four spectral regions, and three different multivariate data analysis methods (PLS-DA, NNet, and xgbTree) have been tested in each region.

For Raman spectra analysis, a single spectral region was analyzed using PLS-DA and xgbTree. Neural networks have also been tested; however, the training algorithm could not converge due to the low sample size.

To assess the predictive discrimination and avoid over-fitting, a 10-time repeated 5-fold cross-validation (50 models) was applied for each method. Since each individual has multiple spectra, folds have been created at the individual level, ensuring that all spectra for a given individual are either in the training or in the test set.

More details are reported in the Supporting Information.

## Results and Discussion

Sixty-three patients with definite and probable ALS according to the revised El Escorial Criteria^27^ and 46 HC subjects were enrolled (Table S1). After the deposition on the IR transparent BaF_2_ support and excess water evaporation, tears displayed a typical fern-like morphology (Figures S1 and S3), surrounded by an amorphous drop border, called coffee ring, which was excluded from FTIR analysis, being its IR absorption intensity too high to obtain reliable results, while measurable by Raman spectroscopy.

Moreover, round granules of a few micrometer diameters were observed and analyzed by FTIR microspectroscopy and found to be enriched in lipids (Figure S3).

FTIR and Raman data were subjected to multivariate analyses (Figure S1) by different methods (PLS-DA, xgbTree, and NNet) with the aim to obtain a high classification performance and to identify the spectral components most relevant for the discrimination. PLS-DA was employed to identify the most important spectral components discriminating between HC and ALS samples since it supports a more straightforward interpretation of variable importance and a greater stability (Table S2).

### FTIR Analysis of Fern-like Morphologies

The following paragraphs show the IR analysis of the tear fern-like morphologies (Figure S3). Due to the complexity of their FTIR spectra, resulting from overlapping absorption of their molecular components, it was necessary to apply second-derivative analysis as a resolution enhancement approach (Figure S2).

The second-derivative spectra were then subjected to multivariate analyses in distinct spectral ranges (3050–2800, 1800–1500, 1500–1200, and 1200–900 cm^–1^) in order to identify the IR response of the main molecular components.

### Analysis of the 3050–2800 cm^–1^ Spectral Range: Lipid and Protein Absorption

**Figure 1:**
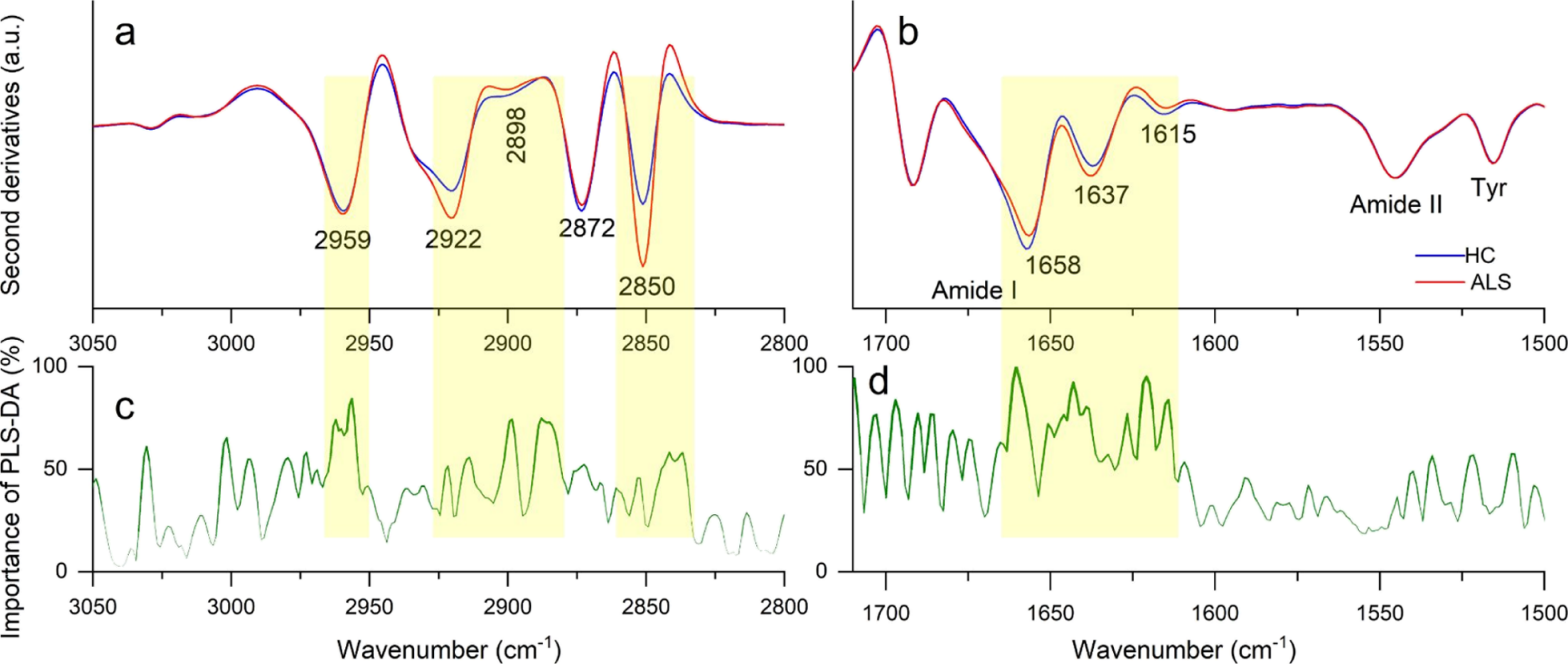
FTIR analysis of fern-like morphologies. Average second-derivative spectra of ALS-positive samples and HCs in the CH_*x*_ stretching range (a) and in the amide I and amide II bands (b). Wavenumber importance (domain 0–100) for the PLS-DA method in the CH_*x*_ stretching range (c) and the amide I and amide II bands (d).

The IR spectra between 3050 and 2800 cm^–1^ show the CH_*x*_ groups of the lipid hydrocarbon chains (Table S2),^28^ with a minor contribution from proteins. As reported in Figure 1a, the second-derivative spectra of ALS and HCs are characterized by four well-resolved bands at ∼2959 cm^–1^ (CH_3_ asymmetric stretching), ∼2922 cm^–1^ (CH_2_ antisymmetric stretching), ∼2872 cm^–1^ (CH_3_ symmetric stretching), and ∼2850 cm^–1^ (CH_2_ symmetric stretching).^28^

Both the CH_2_ bands are more intense in ALS compared to those in HC spectra, indicating longer hydrocarbon chains (Figure 1a) and resulting in an increased CH_2_/CH_3_ ratio (Figure S4), consistent with the reported differences in the lipid chemicophysical properties in intact ALS model astrocytes.^29^

Moreover, all the spectra display a slightly higher absorption at ∼2898 cm^–1^ in HCs compared to that in ALS samples, which is ascribed to the overlapping moieties of lipids (Fermi resonance of CH_2_ symmetric stretching) and of proteins.^30^

**Figure 2:**
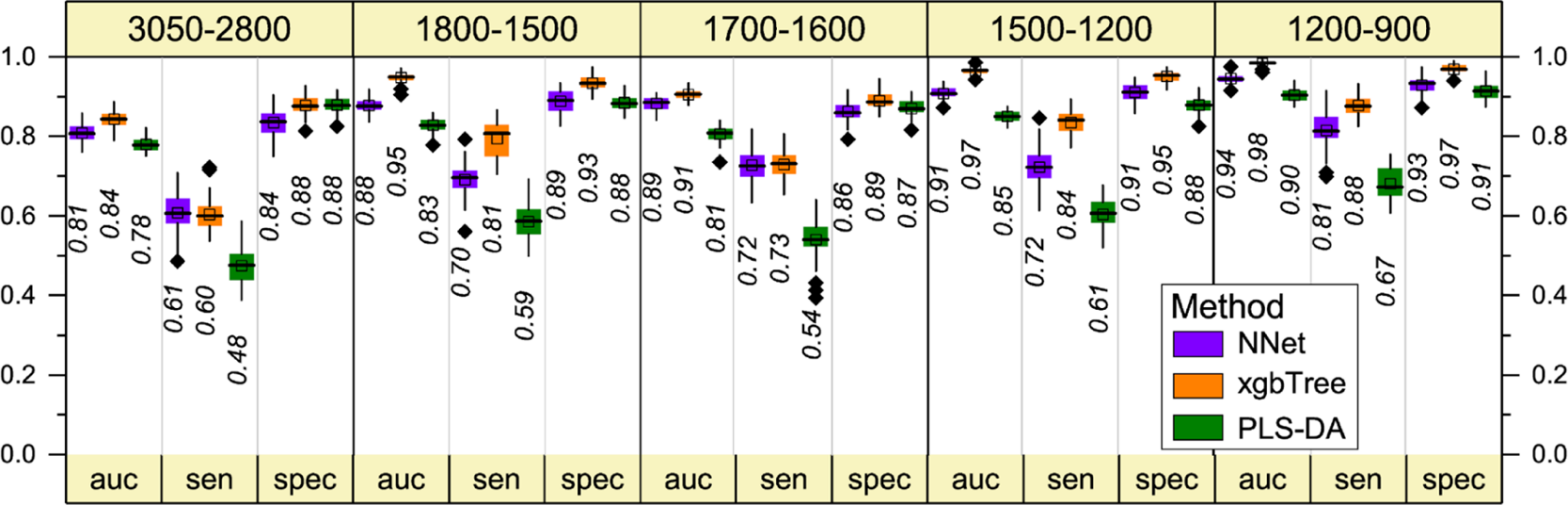
Multivariate analysis of the FTIR second-derivative spectra of fern-like morphologies: overall performance of the NNet, xgbTree, and PLS-DA methods in all the analyzed spectral ranges. In particular, the 5-fold cross-validation resampled area under the curve (auc), sensitivity (sen.), and specificity (spec.) are reported. The black horizontal line within the box is the median, the square within the box is the mean, the box ends show the first (Q1) and third (Q3) quartiles, the lower whisker is computed as the maximum value between the absolute minimum and Q1 – 1.5 × IQR, and the upper whisker is the minimum between the absolute maximum and Q3 + 1.5 × IQR. Here, IQR is the interquartile range computed as Q3–Q1. The values beyond whiskers (outliers) are shown as black diamonds. The median values are also reported.

In the overall discrimination performance of PLS-DA (Figure 2), high values of accuracy (auc of 0.78) and specificity (0.88) have been obtained, together with a low sensitivity value (0.48). A small increase of the discrimination performance has been obtained by NNet and xgbTree analyses (Figure 2). Then, PLS-DA is identified as relevant for the discrimination of most of the CH_2_ and CH_3_ components (Figure 1c), thus confirming that ALS tears are characterized by altered properties of the lipid component. This result hints to lipids as prognostic ALS biomarkers. Alterations of the main lipid classes and circulating lipoproteins in ALS patients have been reported in the literature, highlighting the dyslipidemia as a hallmark of the disease and opening the possibility for therapeutic nutritional intervention.^5,31^

### Analysis of the ∼1700–1500 cm^–1^ Spectral Range: Protein Absorption

Figure 1b reports the second-derivative spectra of tears from ALS and HC samples in the 1700–1500 cm^–1^ spectral range, dominated by the amide I and amide II protein bands (Table S2). The amide I band is characterized by two main components: one at ∼1658 cm^–1^ assigned to α-helices/random coils and one at ∼1637 cm^–1^, mainly due to β-sheets.^32^ The α-helix/random coil component displays a higher intensity in HC compared to that in ALS-positive spectra, where, instead, the β-sheet band is more intense. A component at ∼1615 cm^–1^, of higher intensity in control spectra, is assigned mainly to the amino acid side chains. The higher β-sheet content of ALS samples, also confirmed by the Raman analysis reported below, can be ascribed to differences in the whole protein content and/or to the occurrence of protein misfolding and aggregation in the disease.^1^ Indeed, cytoplasmic inclusions have been found in ALS cellular models^5^ and in motor neurons of ALS patients.^33^

Overall, the above components (Figure 1b) are the most relevant ones in discriminating between ALS and HC samples, as detected by PLS-DA (Figure 1d) with high accuracy and specificity, although with quite low sensitivity (Figure 2). Similar discrimination performance was obtained by NNet and xgbTree analyses, with improved sensitivities compared to PLS-DA (Figure 2).

### Analysis of the 1500–1200 cm^–1^ Spectral Range: Lipid Absorption

**Figure 3:**
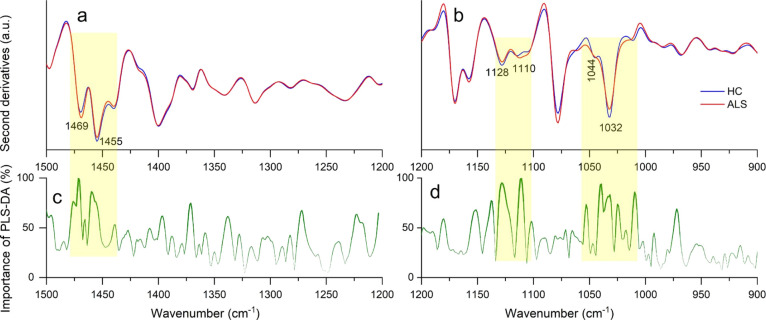
FTIR analysis of fern-like morphologies. Average second-derivative spectra of ALS-positive samples and HCs in the 1500–1200 cm^–1^ (a) and 1200–900 cm^–1^ (b) spectral ranges. Wavenumber importance (domain 0–100) for the PLS-DA method in the 1500–1200 cm^–1^ (c) and 1200–900 cm^–1^ (d) ranges.

The analysis between 1500 and 1200 cm^–1^ (Figure 3a, Table S2) is consistent with the results obtained in the spectral range 3050–2800 cm^–1^. PLS-DA (Figure 3c) indicates as the best discriminating features the ∼1469 cm^–1^ peak (CH_2_ and CH_3_) and the ∼1455 cm^–1^ peak (CH_3_).^28,34^

We should note that also in this case, the accuracy and specificity were high, while the sensitivity resulted to be low (Figure 2).

Interestingly, the overall classification performance obtained by NNet and xgbTree analyses was improved compared to PLS-DA, resulting in accuracies of 0.91–0.97, sensitivities of 0.72–0.84, and specificities of 0.91–0.95 (Figure 2), respectively.

Overall, in agreement with that observed between 3050 and 2800 cm^–1^, these results support the crucial role of lipids as potential ALS biomarkers.

### Analysis of the 1200–900 cm^–1^ Spectral Range

The spectral range between 1200 and 900 cm^–1^ is very complex since it is due to the overlapping absorption of different biomolecules, such as carbohydrates and phosphates, that could reflect protein/lipid glycosylation and phosphorylation, as well as glyco(sphingo)lipid content (Table S2). The second-derivative spectra of ALS and control samples displayed two well-resolved bands, one at ∼1128 cm^–1^, slightly more intense in HCs, and one at ∼1110 cm^–1^, slightly more intense in ALS samples (Figure 3b), both assigned to different phosphate and carbohydrate moieties.^35^ In addition, the absorption at 1032 cm^–1^, with a shoulder around 1044 cm^–1^, is of higher intensity in HC compared to that in ALS tears, and it may be assigned to vibrational modes of carbohydrates and/or glycosphingolipids, as well as to P–O–C in some phospholipids.^35−37^

We tentatively assign these absorptions to glycosylation and phosphorylation of tear lipids and proteins and/or to glyco(sphingo) lipids. The latter have been reported as modulators of ALS pathogenesis and are considered as potential drug targets.^38^ Moreover, a very recent study reports excess sphingolipid biosynthesis as a fundamental metabolic mechanism for ALS.^39^ In agreement with this result, the analysis of lipid granules (Figure S7b, d) identifies, among the components relevant for the discrimination, the 1065 cm^–1^ peak, which has been assigned to glycolipids.^40^ In addition, the major constituents of tears are glycoproteins (e.g., IgA, lactoferrin, and lacritin),^41^ and protein glycosylation has been found altered in patients affected by different neurodegenerative disorders, including Huntington’s, Alzheimer’s, and ALS diseases.^42^

The overall classification performance of PLS-DA in this spectral range was satisfactory (Figure 2), and, as observed for the other spectral ranges, the xgbTree and NNet analyses achieve an improvement not only in accuracies (0.94–0.98, respectively) and specificities (0.93–0.97) values but also in sensitivity (0.81–0.88).

### Raman Analysis of Tears

**Figure 4:**
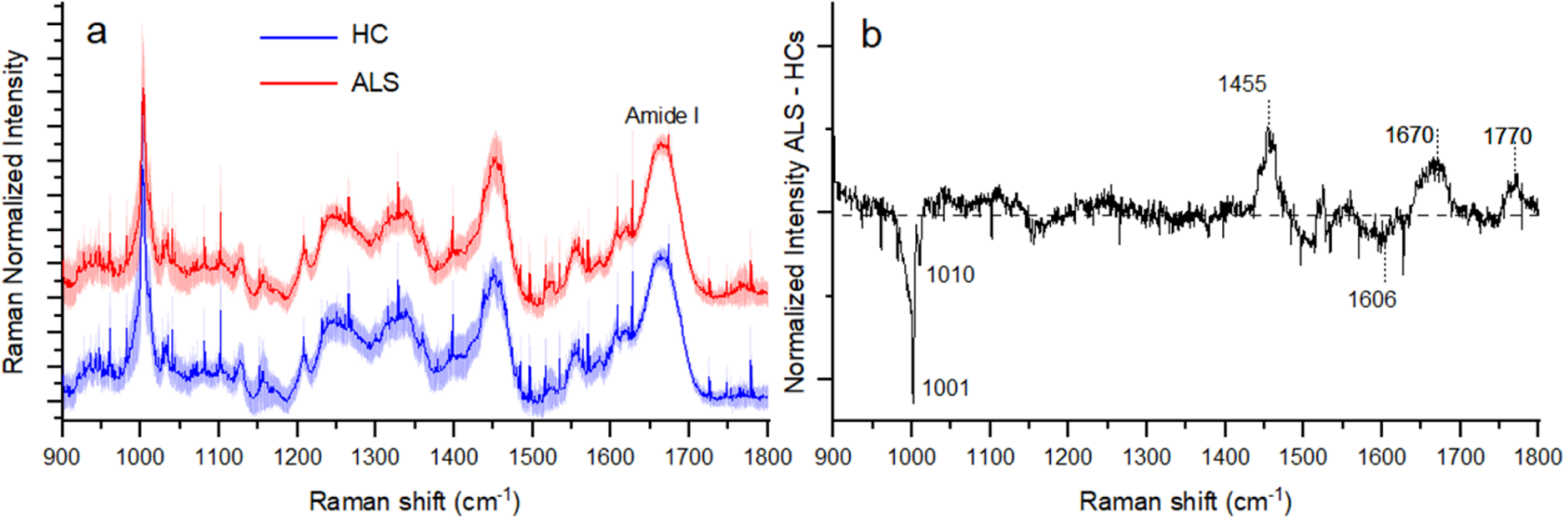
Comparison of the mean Raman spectra obtained by considering all the measured tears from ALS patients and HCs (a). The shadowed area refers to the standard deviation of the data. (b) Spectrally resolved differential average Raman spectra of the two investigated groups.

Raman investigation has been performed on tear samples prepared by the same procedure employed for the FTIR studies since the BaF_2_ substrate does not interfere with the analysis (Figure S8). Figure 4a shows well-defined peaks in the 900–1800 cm^–1^ range and demonstrates that the average Raman spectra of both ALS and HC groups are consistently dominated by a rather narrow feature at 1000 cm^–1^ and by additional broader peaks located above 1200 cm^–1^. The stronger Raman band, being centered at ∼1003 cm^–1^, is likely given by urea^43^ with contributions from phenylalanine.^44^ The protein Raman response of tears is detected in the amide I band due to the stretching vibrations of the backbone C=O groups, with peaks at 1657 cm^–1^ (α-helices) and 1670 cm^–1^ (β-sheets).^44^ The band at ∼1250 cm^–1^ is assigned to amide III. Finally, the weak feature at ∼1770 cm^–1^ is ascribed to the C=O stretching of lipids.^45,46^

**Figure 5:**
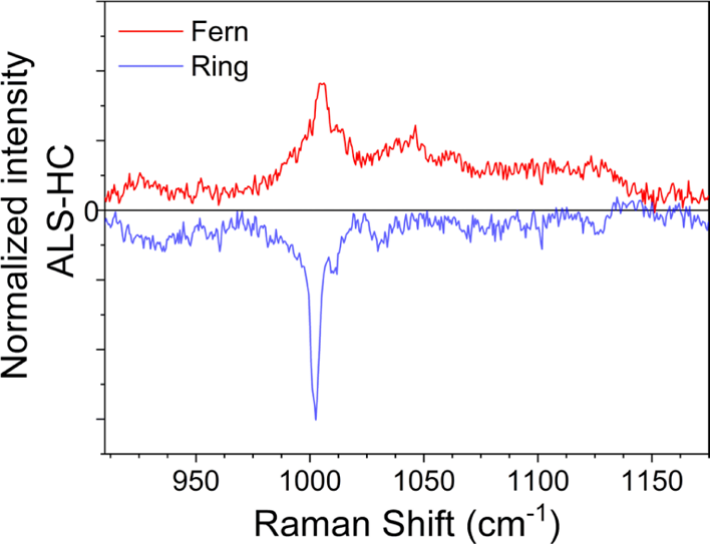
Differential average Raman spectra of ALS patients and HCs measured in the fern-like patterns at the center of the dried drop (red line) and in the coffee ring (blue line).

Figure 4b shows that the differential average spectra of the two sample classes allow identifying ALS-specific molecular markers. Notably, ALS patients demonstrate on average a marked reduction of the Raman band at ∼1000 cm^–1^. The relative minima in this spectral region (Figure 4a) occur at ∼1001 cm^–1^ and 1010 cm^–1^ and are echoed by the peak at ∼1606 cm^–1^. This eventually heralds a remarkable global reduction of the phenylalanine level in the tear fluid caused by the disease. Our Raman data thus suggest a striking malfunctioning of the metabolism of amino acids. Such phenomena can cause alterations in the neurotransmitter regulation, which are known to result in severe neurological dysfunctions.^47^ An enhanced phenylalanine consumption harbors the occurrence of an energy crisis in ALS pathogenesis. This also leads to the possible consequence that muscle weakness and paralysis might emerge during the disease progression as a result of the back action triggered by the body to counterbalance the phenylalanine deficit. Such findings support the observation that ALS can be fully regarded as a metabolic neurodegenerative disease.^48^ Further insights can be obtained by mapping the average Raman band at about 1000 cm^–1^ in the protein-enriched peripheral ring and in the salt-rich region at the center of the tear drop. Differential spectra shown in Figure 5 of ALS patients and HCs measured at the center (red line) and at the border (blue line) of the dried drop are illuminating. While some of the vibrational features originally present in Figure 4 cancel out in the differential data, being constant between the two sets of data, two well-pronounced negative dips develop at 1001 and 1010 cm^–1^ in Figure 5. Such features further corroborate the deficiency of phenylalanine vibrations in the external ring. On the contrary, the fern-like patterns show a complete sign reversal of the differential spectrum, and the positive maximum at ∼1003 cm^–1^ (Figure 5) demonstrates a surge of urea in ALS patients.

Our average Raman measurements show in the ALS group a distinct increase of the spectral weight of the phonon modes at ∼1670 cm^–1^ (protein β-sheet structures) and 1770 cm^–1^ (C=O stretching of lipids). In particular, in agreement with the FTIR results reported above, these findings confirm the enrichment in β-sheet structures in tears from ALS patients compared to that from HCs.

To further corroborate our findings, in the following, we discuss the multivariate analysis of the Raman data.

**Figure 6:**
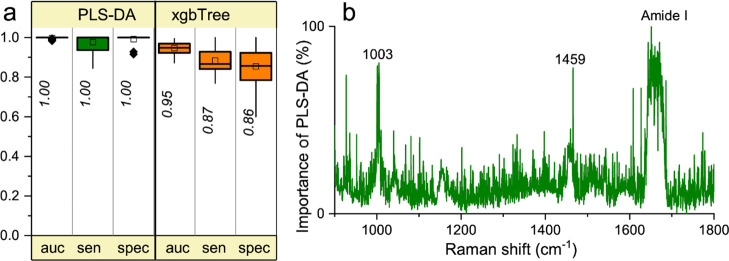
Multivariate analysis of Raman spectra. (A) Overall performances of PLS-DA and xgbTree methods in the 900–1800 cm^–1^ spectral range. In particular, the resampled area under the curve (auc), sensitivity (sens.), and specificity (spec.) are reported as in Figure 2. (B) Wavenumber importance (domain 0–100) for PLS-DA method in the 900–1800 cm^–1^ spectral range.

Figure 6 shows the classification of tears from ALS patients and HCs carried out using PLS-DA after having normalized the spectra for the area of the amide I band. We also validated the classification by means of the xgbTree method (Figure 6a) and by the alternative intrinsic reference offered by the peak at about 1000 cm^–1^ (data not shown). The PLS-DA analysis of the Raman spectra shows a high classification accuracy with a specificity and sensitivity of ∼100%. The relative weight of the important coefficients in the whole 900–1800 cm^–1^ range unveils the most important spectral components discriminating between ALS patients and HCs. Such notable Raman modes can be observed in Figure 6b as they distinctly emerge from the noisy background. According to the PLS-DA method, phenylalanine and amide I are the most important signatures for the classification, thus strengthening the observation that metabolic dysfunctions and alterations of the whole protein structures and/or content might play a crucial role in this neurodegenerative disease. The consequences of such impairment reflect themselves in the tear fluid and provide us with a convenient prospective means in quest for identifying ALS development and progression. In line with the PLS-DA data, the ratio between amide I and phenylalanine band area (Figure S9) was found to be higher for tears from ALS patients compared to that from HCs.

The xgbTree method, too, shows a high classification performance (0.95) with a sensitivity of 0.87 and a specificity of 0.86.

## Concluding Remarks

Vibrational spectroscopies are gaining increasing attention in clinics because they provide a fast and label-free way to a holistic picture of biological samples. Finding non-invasive diagnostic tools for neurodegenerative disorders is a highly desirable goal for clinicians and a priceless benefit for patients. In this perspective, we applied FTIR and Raman microspectroscopies, coupled to multivariate analysis, for the characterization of tears from ALS patients and HCs, as an easily accessible diagnostic method with the notable potential of providing unmatched insights into pathology. Our approach allows discriminating between diseased and healthy samples with a high overall performance and does not require molecular labeling nor expensive and unpractical identifications of molecular biomarkers. Furthermore, multivariate analysis discloses the spectral components responsible for the discrimination, which can then be assigned to specific biomolecule classes, a result that is highly desirable for a multifactorial pathology such as ALS.

In particular, these vibrational analyses highlight differences in the protein content and structures in ALS and HC tears. The former are characterized by a higher β-sheet content compared to the latter, suggesting alterations in protein conformation and/or tear composition and supporting a role of protein misfolding and aggregation in the disease.

It is also found that ALS tears are characterized by a higher CH_2_/CH_3_ ratio compared to HCs, thus indicating changes in the lipid physicochemical properties, in agreement with the crucial role of dyslipidemia as a hallmark of the disease. Furthermore, the results highlight a reduction of the phenylalanine level in ALS tears, suggesting a malfunctioning of the metabolism of amino acids.

Looking ahead, the combined use of such spectroscopies and omics approaches is expected to provide composite biomarkers with improved diagnostic performance and to allow a better comprehension of multifactorial pathologies and other NDs. Overall, our proof-of-concept work demonstrates that vibrational spectroscopy characterization of tears could represent a powerful tool for neurodegenerative disorder diagnosis, whose specificity needs to be evaluated on a large cohort of patients affected by different diseases sharing common features with ALS. The correlation of spectroscopic data with molecular markers, such as those derived from multi-omics approaches, and the possibility to discriminate among ALS-mimicking diseases will represent an important step for the translation into clinic. This kind of study has been performed for neurodegenerative diseases on other biofluids.^49−51^ We believe that the unique features introduced by our method can be key enabling factors for the future introduction of tear vibrational spectroscopy into clinical practice and for the implementation of hand-held devices to be utilized in point-of-care diagnostics. The translation into the clinics would then require the application of automated and high-throughput devices, a major thrust of current research in the field.^52,53^

## Supporting Information Available

Overview of the experimental workflow; additional FTIR analyses; additional Raman analyses; methods, results, and discussion for the FTIR characterization of lipid granules; more details on the Raman analyses (experimental details and assignment of Raman bands); descriptive analysis of ALS patients and HCs; and assignment of the relevant IR components (PDF)
