# Supplementary material for: Tear-Based Vibrational Spectroscopy Applied to Amyotrophic Lateral Sclerosis
Source: Anal Chem. 2021 Dec 14;93(51):16995–7002. doi: 10.1021/acs.analchem.1c02546 (PMC8717331; doi:10.1021/acs.analchem.1c02546)
Supplement: Supplementary file 1 — ac1c02546_si_001.pdf [file ac1c02546_si_001.pdf]

Supporting Information for

Tear-based vibrational spectroscopy applied to amyotrophic lateral sclerosis

Diletta Ami[1], Alessandro Duse[2,3], Paolo Mereghetti[4], Federica Cozza[3,5], Francesca Ambrosio[1], Erika Ponzini[2,3], Rita Grandori[1], Christian Lunetta[5,6], Silvia Tavazzi[2,3], Fabio Pezzoli[2]*, Antonino Natalello[1]*

[1)]Department of Biotechnology and Biosciences, University of Milano-Bicocca, Piazza della Scienza 2, 20126, Milano, Italy.
[2)] Department of Materials Science, University of Milano-Bicocca, Via R. Cozzi 55, 20125, Milano, Italy.
[3)] COMiB Research Centre in Optics and Optometry, Via R. Cozzi 55, 20125, Milano, Italy.
[4)] Bioinformatics Consultant, 15061, Arquata Scrivia, Italy.
[5)] NEuroMuscular Omnicentre (NEMO), Serena Onlus Foundation, Piazza Ospedale Maggiore 3, 20162, Milano, Italy.
[6)] NEMO Lab, Piazza Ospedale Maggiore 3, 20162, Milano, Italy.

*Correspondence: fabio.pezzoli@unimib.it, Department of Materials Science, University of Milano-Bicocca, via R. Cozzi 55, 20125 Milano, Italy, Phone:+39 02 6448 5175.

antonino.natalello@unimib.it, Department of Biotechnology and Biosciences, University of Milano-Bicocca, P.zza della Scienza 2, Milano, Italy, Phone:+39 02 6448 3459.

This PDF file includes:

Figures S1 to S9

Supplementary materials and methods

Results and Discussion for the FTIR characterization of lipid granules

Raman Analyses

Table S1 and Table S2.

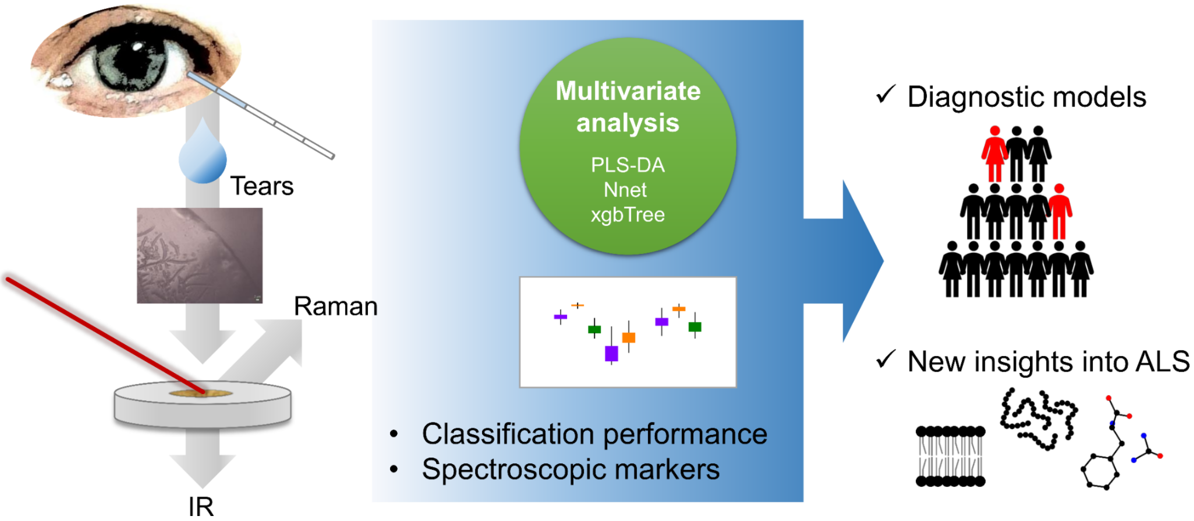

Figure S1. Overview of the experimental workflow. Tears are non-invasively collected from ALS positive patients and healthy controls (HCs) and drop-casted onto a BaF_2 window. When allowed drying under controlled conditions, a net flux of solution is driven towards the drop edge by the so-called coffee-stain effect[1]. This spontaneous process yields the peripheral deposition of the colloids and the formation of an external amorphous ring, where proteins tend to segregate[2]. At the centre of the drop, instead, a dendritic crystallisation occurs in fern-like patterns, where proteins, urea and salts accumulate preferentially [2,3]. Consequently, drop-coating is particularly advantageous, because it spontaneously results in analyte partitioning of the fluid. After drop-casting, samples are analysed through FTIR and Raman spectroscopies and the classification carried out by means of dedicated multivariate analyses, in particular partial least square discriminant analysis (PLS-DA), neural networks (NNet), and extreme gradient boosting (xgbTree). Owning to this process flow, we can identify the most significant spectral changes responsible for the discrimination between ALS positive and HC tears.

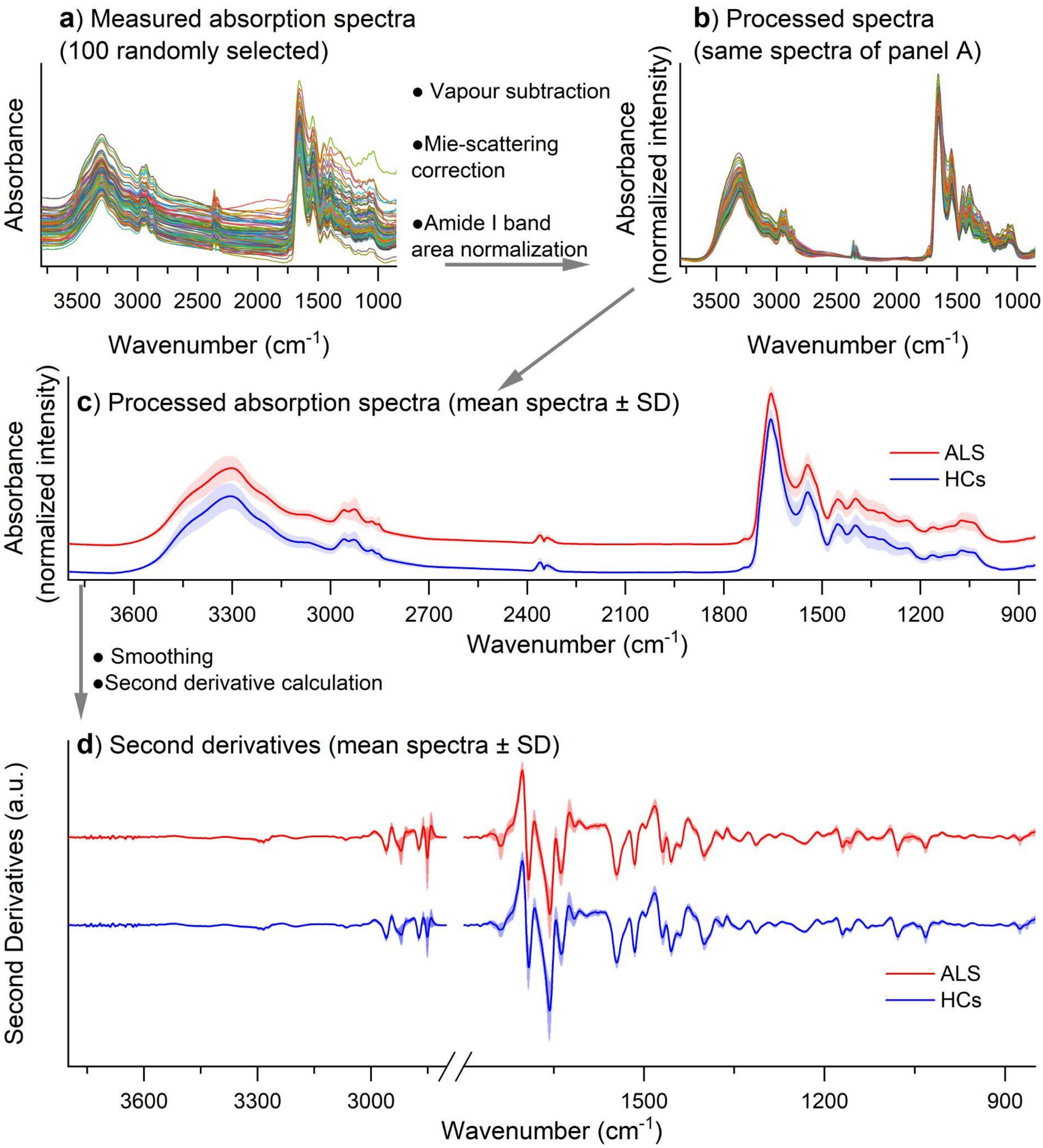

Figure S2. Overview of the FTIR spectra analysis. For a few measured spectra it was necessary to perform a correction for the vapour absorption. In particular, the presence of small positive or negative very sharp peaks observed at specific and well known wavenumbers was taken as an indication of vapour interference. Several peaks in the 1750-1500 cm^{-1} spectral range - in particular the ~ 1653 cm^{-1} peak - were considered. The vapour spectrum was subtracted from the sample spectra until a smooth profile around these peaks was observed. After water vapour subtraction, the spectra were corrected for Mie scattering using the algorithm developed by Bassan and colleagues [4], where the spectral range (originally between 4000-1000 cm^{-1}) was extended in our laboratory down to 800 cm^{-1}. For a better comparison, the corrected spectra were normalized at the Amide I band area and the second derivative analysis was performed (after a 13-point smoothing of the measured

spectra) by the Savitzky-Golay method (3rd polynomial, 9 smoothing points), using the GRAMS/32 software (Galactic Ind. Corp., Salem, NH, USA).

One hundred randomly selected raw spectra were reported in (a) and after the correction procedures in (b).

The average absorption (c) and second derivative (d) spectra obtained from the fern-like morphology of all the analysed tear samples are also reported.

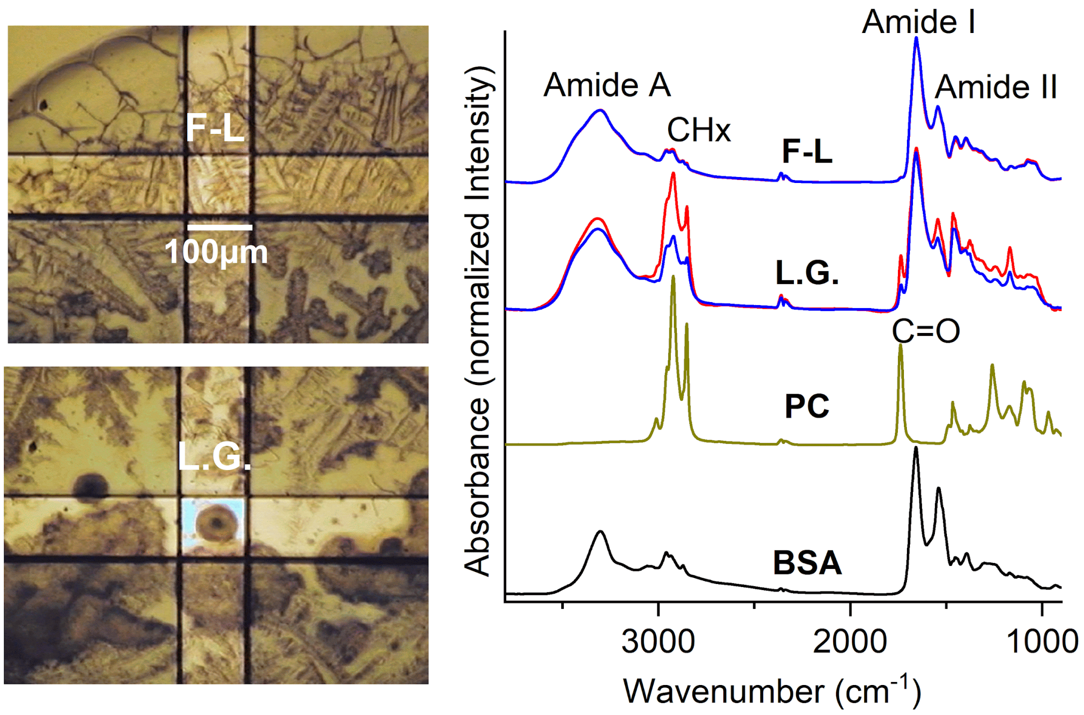

Figure S3. Tear morphologies. Optical images of tear samples after deposition on a BaF_2 IR window and water evaporation. The top image displays fern-like morphologies, below the lipid granules. On the right, the average FTIR absorption spectra of fern-like morphologies (F-L) and lipid granules (L.G.) of tears from ALS positive (red spectra) and HC (blue spectra) subjects are shown. For comparison, the absorption spectra of phosphatidylcholine (PC) and of bovine serum albumin (BSA) are reported as, respectively, phospholipid and protein standard. The assignment of the main bands to protein (Amide A, I, II) and lipid (CH_x and C=O) moieties is indicated.

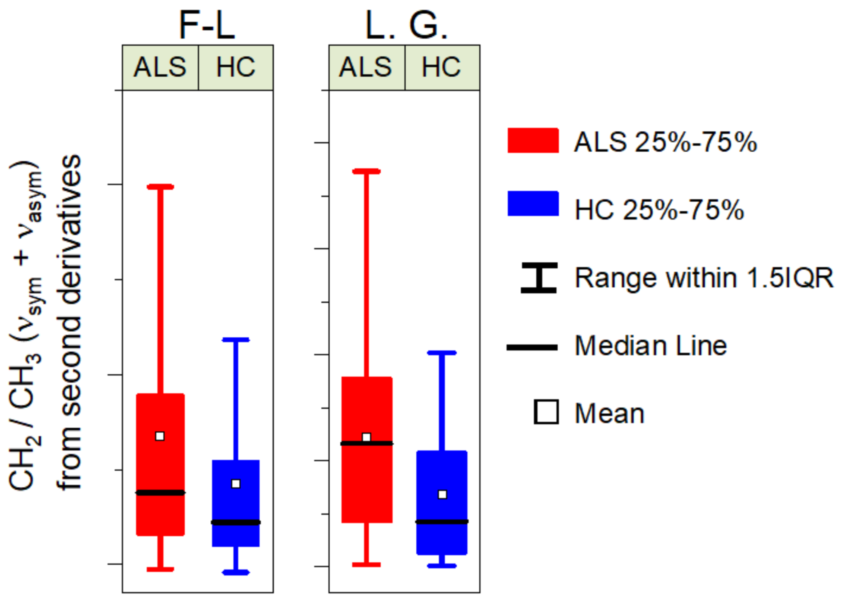

Figure S4. CH_2/CH_3 intensity ratio from second derivatives of the FTIR spectra of tears from ALS positive and HCs. F-L, fern-like morphologies; L.G., lipid granules.

Before performing the T-test analysis of the differences between the ALS and HC groups, the normality assumption and the variance homogeneity were verified by the Shapiro-Wilk test and the Levene test, respectively. Since the normality assumption and the homogeneity of variance could not be verified in all cases, the non-parametric test Wilcoxon rank sum test was also performed. In all cases, the two populations have different means at the significance level of 0.01.

F-L: T-test P-value 1.928e-12; Wilcoxon rank sum P-value 8.338e-15;

L.G.: T-test P-value 0.0006072; Wilcoxon rank sum P-value 0.001379.

SUPPLEMENTARY MATERIALS AND METHODS

ALS patients and healthy controls

All the demographic and clinical features were collected, including the Amyotrophic Lateral Sclerosis Functional Rating Scale–revised (ALSFRS-r) total and domain scores, the disease duration expressed as the time from onset to tear collection, the disease progression rate calculated as (48 minus ALSFRS-r total score)/disease duration, the site of onset (limb or bulbar), and the use of invasive ventilation (Table S1).

The following inclusion criteria were applied for patients enrollment: diagnosis of definite/probable ALS (El Escorial Diagnostic Criteria); age 18-90 years.

The following exclusion criteria were applied: severe clinical conditions limiting the compliance to the study; the presence of other severe comorbidities; patients taking drugs (e.g. anticholinergic) that could negatively influence the tear sampling. Compared to the ALS group, healthy subjects were matched by age and gender.

The study obtained the University (july 2019, n. 484) and NEMO-Milano Area 3 (september 2019, n. 479-092019) ethics committee's approvals.

Additional information on the performed multivariate analyses

Multivariate analysis has been performed using R version 3.6.3 (https://www.R-project.org/, https://cran.r-project.org/package=nnet (Brian Ripley, William Venables, 2021), https://cran.r-project.org/package=caret (Max Kuhn, Jed Wing, Steve Weston, Andre Williams, Chris Keefer, Allan Engelhardt, Tony Cooper, Zachary Mayer, Brenton Kenkel, R Core Team, Michael Benesty, Reynald Lescarbeau, Andrew Ziem, Luca Scrucca, Yuan Tang, Can Candan, Tyler Hunt, 2021)).

PLS-DA is a widely used multidimensional linear regression method, which is a variant of the classical partial least square method when the dependent variable is categorical [5]. NNet is a single hidden layer feed-forward neural network [6] implemented in the R package "nnet" version 7.3-13. Neural network parameter size (number of units in the hidden layer) and decay (regularization parameter to avoid over-fitting) have been tuned using a tuning grid with *size* varied from 1 to 5 by 1 and *decay* varied from 0.05 to 0.3 by 0.05. Spectra have been center-scaled (normalized in order to have 0 mean and variance 1) to allow faster convergence of the training algorithm [7].

The xgbTree belongs to the family of the boosting methods, i.e. classification methods that produce classification models from an ensemble of weak classifiers, classification trees in this case. The extreme adjective refers to the algorithmic tricks implemented in order to increase the computational performances [8,9]. In all training cases, the XgbTree parameters *eta* (shrinking parameters used to avoid over-fitting) and *max-depth* (maximum depth of a tree) have been set to 0.1 and 6 respectively.

In order to assess the predictive discrimination and avoid over-fitting, for each method a 10-time repeated 5-fold cross-validation was applied. So for each method 50 models were trained. Since each individual has multiple spectra, folds have been created at the individual level, ensuring that all spectra for a given individual are either in the training or in the test set. More specifically, having N individual each with m_N spectra, on every round of cross-validation, the individuals have been partitioned into 5 folds. Four folds (containing N*4/5 individuals) have been used to train the model and the remaining fold (containing N*1/5 individuals) was used to test the model. Folds are complementary (i.e. no repeated individuals in different folds) and the individuals are randomly chosen. The training of the model is repeated five times, each time varying the test partition. The 5-fold cross-validation is then repeated 10-times in order to lower the risk of partition-dependent artifacts. The best model has been selected using the "one standard error rule". In this case, the model with the best performance value is identified and, using resampling, we can estimate the standard error of performance. The final model used was the simplest model within one standard error of the (empirically) best model[10]. As a performance measure, the area under the curve (auc) was used. The auc is computed from the receiver operating characteristic curve (ROC curve) that is created by plotting the true positive rate (i.e. sensitivity) against the false positive rate (i.e. specificity) at various threshold (value that discriminates between positive and negative outcome) settings. When using normalized units, the auc is equal to the probability that a classifier will rank a randomly chosen positive instance higher than a randomly chosen negative one (assuming "positive" ranks higher than "negative") [11]. For all trained models the sensitivity and specificity have been also computed. The sensitivity is computed as true positive / (true positive + false negative), while the specificity as true negative / (true negative + false positive). For the PLS-DA method the variable importance measure here is based on weighted sums of the absolute regression coefficients[10].

Neural networks, despite reaching a high predicting power, can be unable to find stable relations between explanatory variables and dependent variables[12]. Similarly, in boosting trees a correct

interpretation of variable importance may also be an issue [13]. PLS-DA is a linear method which may not reach a predicting power comparable to NNet or xgbTree. However, because of its linear nature and its intrinsic robustness it allows a more straightforward interpretation of variable importance and a greater stability [14]. For this reason, we decided to rely on the PLS-DA analysis to identify the most important spectral components in the discrimination between tears from HCs and ALS patients (Table S2).

INFRARED ABSORPTION OF LIPID GRANULES

After tear deposition on a BaF_2 IR window and excess water evaporation, besides the fern-like morphologies we identified also round granules (Fig. S3) that resulted enriched in lipids, as clearly indicated by their FTIR spectra (Fig. S3). In consideration of the observed typical dimension of lipid granules (several tens of microns), they likely formed as a consequence of water evaporation. These granules were analyzed separately from the fern-like morphologies. These structures displayed different spectral features in ALS positive and healthy controls, further supporting that lipids deserve to be studied as potential biomarkers of the pathology. As shown for the fern-like structures, we analyzed the second derivative spectra - with the support of PLS-DA, xgbTree and NNet - in different spectral ranges.

Analysis of the 3050-2800 cm^{-1} spectral range: lipid absorption

As reported in Fig. S5a, the IR second derivative spectra of lipid granules from ALS positive and HC tears between 3050 and 2800 cm^{-1}, mainly due to the CHx group of the lipid hydrocarbon chains, display different spectral features[15]. In general, the relative lipid content appears significantly more intense in the positive samples compared to healthy controls, and characterized by longer hydrocarbon chains. As indicated in Fig. S5c, the PLS-DA identifyed as relevant for discrimination between the two classes the bands at ~ 2965 cm^{-1} and ~ 2871 cm^{-1}, both assigned to CH_3 groups, with a contribution of the CH_2 component at ~ 2850 cm^{-1} (Table S2). The PLS-DA, xgbTree and NNet reported high accuracy and specificity values, while the sensitivity was lower, indicating a significant number of false negatives (see Fig. S6 for details).

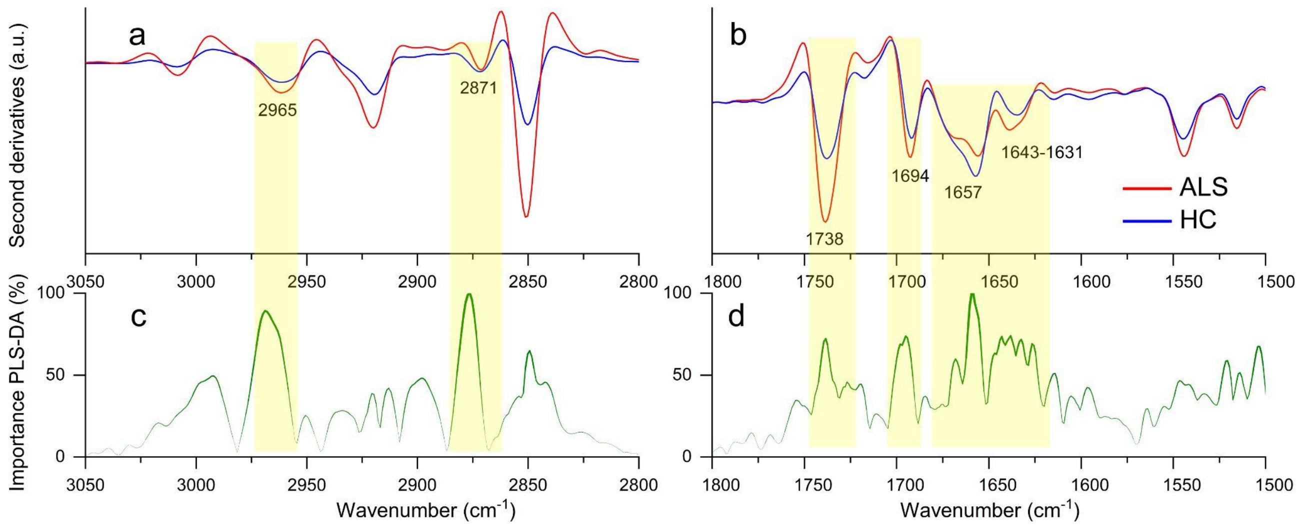

Figure S5. Average second derivative spectra of ALS positive samples and HCs in the CHx stretching range (a) and in the Amide I and Amide II bands (b). Wavenumber importance (domain 0-100) for PLS-DA method in the CHx stretching range (c) and the Amide I and Amide II bands (d).

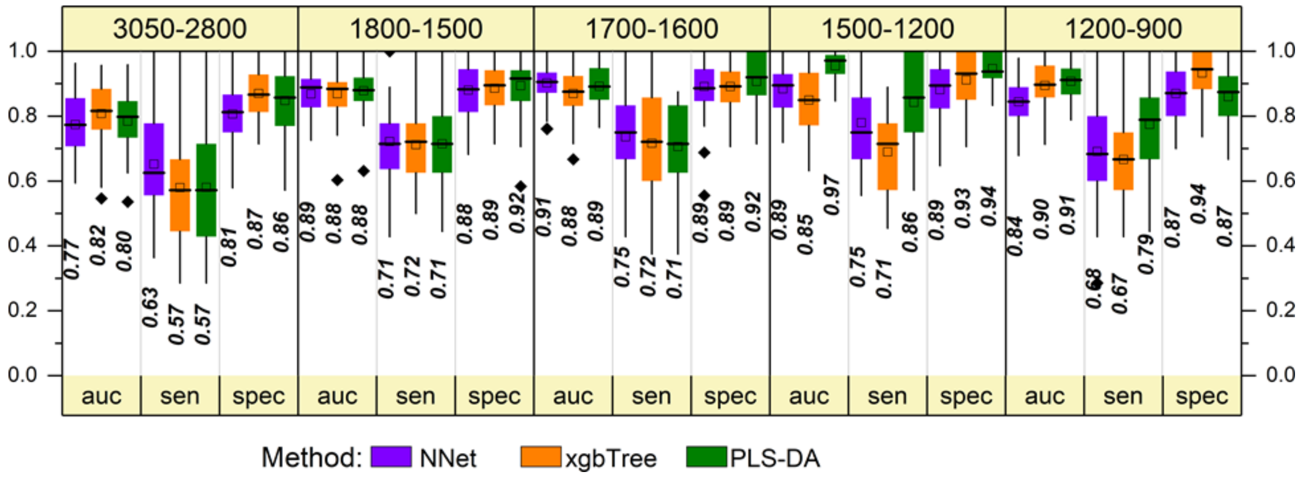

Figure S6. Multivariate analysis of lipid granules FTIR second derivative spectra. Overall performances of NNet, xgbTree and PLS-DA methods in all the analyzed spectral ranges, as in Figure 2.

Analysis of the 1800-1500 cm^{-1} spectral range: protein absorption

In Fig. S5b, we reported the lipid granule second derivative spectra of ALS positive and HC tears, between 1800-1500 cm^{-1}, where the proteins of these structures mainly absorb[16–18]. The spectra are characterized by a few well resolved bands of different intensity in the two classes. These

components, almost all relevant to discriminate between ALS positive and negative controls, as indicated by PLS-DA analysis (Fig. S5d, Table S2), are: ~ 1657 cm^{-1}, mainly assigned to α-helix and random coil structures, of higher intensity in negative controls; between ~ 1643-1631 cm^{-1} and at ~ 1694 cm^{-1}, mainly due to β-sheet structures [16–18], both of higher intensity in ALS positive samples, and ~ 1738 cm^{-1} - mainly assigned to the ester C=O groups[15] - that appears of higher intensity in ALS positive samples. Interestingly, the PLS-DA classification performance (Fig. S6) is good, with accuracy 0.88-0.89, sensitivity 0.71, and specificity 0.92. As reported in Fig. S6, the classification performances of xgbTree and NNet methods were similar to those obtained by PLS-DA.

Analysis of the 1500-1200 cm^{-1} spectral range: lipid absorption

The PLS-DA analysis of the spectral range between 1500-1200 cm^{-1} (Fig. S7a,c and Table S2) indicated as the most important component for the discrimination between ALS positive and HC tears the band at ~ 1470 cm^{-1} (Fig. S7c), which is mainly due to the hydrocarbon chain CH_2 and CH_3 groups [15,19]. As it can be seen, this component displays a significant higher intensity in ALS positive tears compared to HCs (Fig. S7a), in agreement with the results obtained between 3050-2800 cm^{-1}. Noteworthy, the classification performance of this spectral range is high, as reported in Fig. S6.

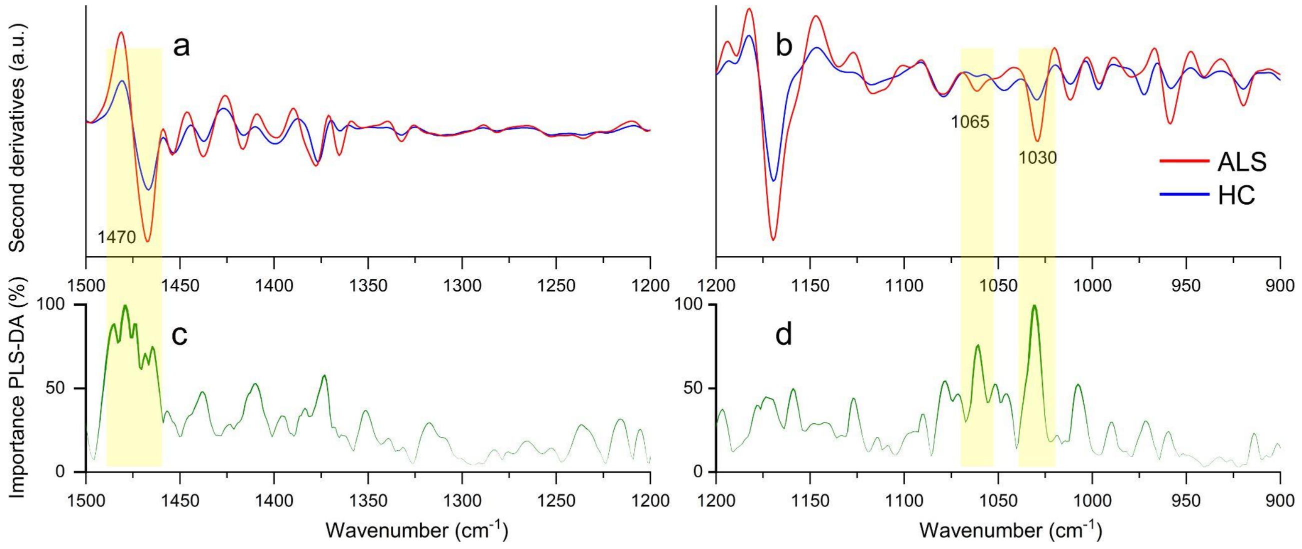

Figure S7. FTIR analysis of lipid granules. Average second derivative spectra of ALS positive samples and healthy controls in the 1500-1200 cm^{-1} (a) and 1200-900 cm^{-1} (b) spectral ranges. Wavenumber importance (domain 0-100) for PLS-DA in the 1500-1200 cm^{-1} (c) and 1200-900 cm^{-1} (d) ranges.

Analysis of the 1200-900 cm^{-1} spectral range

As discussed for the fern-like structures, the spectral range 1200-900 cm^{-1} is very complex, being dominated by the overlapping contributions of different vibrations ascribable mainly to carbohydrates and phosphates.

In particular, the PLS-DA (Fig. S7d) reported as the most significant spectral differences between ALS positive and HC tears - with a satisfactory classification performance (Fig. S6) - the components at ~ 1065 cm^{-1} (C-O, C-C mainly from glyco(sphingo)lipids; P-O-C mainly of phospholipids)[20-21], negligible in healthy controls, and at ~ 1030 cm^{-1} (C-O/C-C carbohydrates and P-O-C in some phospholipids)[20-22], of higher intensity in ALS positive tears. The classification performance obtained in this spectral region by the three multivariate methods is reported in Fig. S6.

RAMAN ANALYSES

Experimental details

The investigated spectral range was 900-1800 cm^{-1} and was obtained by collating neighboring spectral windows, with a 60 x 5 seconds time of measure each. For every sample, different spectra were measured at various locations to account for concentration gradients and inhomogeneities caused by proteins and lipid segregation during water evaporation. At visible excitations, fluorescence is typically reduced in dried tears compared to other biological environments, i.e. tissues and liquids, however, Raman spectra with an excessive luminescence-related background were discarded from the successive analysis to avoid invasive numerical treatment of the raw data and consequent spurious results.

During the measurements, an automated algorithm was applied to remove cosmic ray spikes using the native software of the Raman spectrometer. Baseline correction and area-under-the-curve normalization were performed using the Bioinformatics Toolbox™ of Matlab and functions like msbackadj. Comparable results were obtained by normalizing the spectra for both the Amide I and phenylalanine bands.

Assignment of Raman bands

The peaks observed in Figure 4a of the main text at 1001 cm^{-1}, 1010 cm^{-1} and minor structures in the 1030-1040 cm^{-1} range can be assigned to aromatic amino acids, predominantly phenylalanine [23]. Moreover, the presence of tryptophan is explicitly pointed out by weaker peaks at 960 and 981 cm^{-1} [23]. The spectral features around 1160 cm^{-1} are due to C-C stretching in fatty acids [24]. The presence of methylene groups of lipids is evidenced by a twisting mode at 1298 cm^{-1} and a skeletal stretching at 1127 cm^{-1}, although stretching of the C-N bond in peptide groups can also contribute to the latter.

It should be noted that C=C stretching in cholesterol esters and fatty acids can contribute to the Amide I band at about 1660 cm^{-1} [24]. The band at ~1250 cm^{-1} is assigned to the Amide III, i.e. N-H bending vibrations of the peptide groups, while the broad but intense feature in the 1320 -1340 cm^{-1} range is due to stretching vibrations of the aliphatic side-chains [23].

C-H vibrations in proteins and lipids, like the methylene scissoring motion, contribute to the wide band observed at about 1450 cm^{-1} [23,24], whereas the vibrational mode at about 1550 cm^{-1} arises from ring vibrations of tryptophan [23]. Finally, the weak feature at ~1770 cm^{-1} is ascribed to the C=O stretching of lipids [24,25].

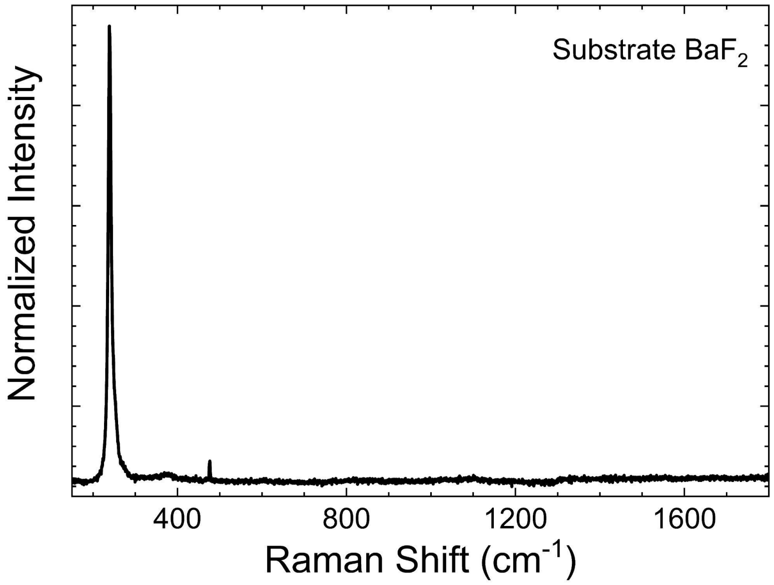

Figure S8. Raman spectrum of the bare BaF_2 substrate. The characteristic Raman mode of BaF_2 occurs as a sharp peak at 243 cm^{-1}. Consequently, the substrate does not interfere with the investigation of proteins and lipids composing the biofluid since their atomic mass and bond stiffness result in higher energy phonons.

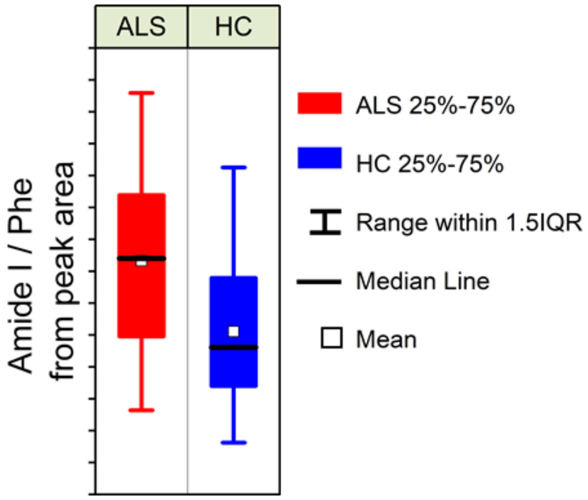

Figure S9. Ratio of Amide I/Phe peak area from Raman spectra of ALS and HC tears. The statistical analysis of the differences between the ALS and HC groups was performed as described in Figure S4. The two populations have different means at the significance level of 0.01:
T-test P-value 7.995e-07; Wilcoxon rank sum P-value 4.12e-06.

Demographic and clinical characteristics of ALS patients	FTIR (n = 57)	Raman (n = 20)
Age, years	66 [55 – 73]	64 [50 – 72]
Sex, male	36 (63%)	12 (60%)
Disease duration, months	36 [21 – 69]	35 [15 – 71]
Disease progression rate, ALSFRS-r score unit/months	0.51 [0.30 – 0.89]	0.56 [0.24 – 0.99]
Gene mutation	5 (9%)	2 (10%)
Site of onset, limb	46 (81%)	15 (75%)
IV at tear collection	7 (12%)	1 (5%)
ALSFRS-r total score	24 [9 – 33]	21 [18 – 37]
ALSFRS-r bulbar subscore	9 [4 – 11]	9 [5 – 10]
ALSFRS-r motor subscore	6 [2 – 16]	11 [3 – 16]
ALSFRS-r respiratory subscore	3 [2 – 12]	9 [2 – 11]
**Demographic characteristics of HCs**	**FTIR (n =36)**	**Raman (n = 18)**
Age, years	57 [49 – 63]	54 [45 – 65]
Sex, male	25 (69%)	11 (61%)

Table S1. Descriptive analysis of ALS patients and HCs. Data are n (%) or median [interquartile range] values. Abbreviations: ALSFRS-r, Amyotrophic Lateral Sclerosis Functional Rating Scale – Revised; IV, Invasive Ventilation.

**Peak position (cm^{-1}) from IR second derivative spectra**	**Assignment**	**Ref.**
2965, 2959	ν-asym CH_3 mainly from lipid hydrocarbon chains	15
2922	ν-asym CH_2 mainly from lipid hydrocarbon chains	15
2898	Lipid and protein moieties (mainly CH_2)	26
2871	ν-sym CH_3 mainly from lipid hydrocarbon chains	15
2850	CH_2 ν-sym mainly from lipid hydrocarbon chains	15
1738	C=O mainly from esters	15
1694	Proteins, β-sheets	16–18
1658, 1657	Proteins, α-helices/ random coils	16–18
1643-1631, 1637	Proteins, β-sheets	16–18
1615	amino-acid side chains	16
1470, 1469	δ CH_2/CH_3 mainly from lipid hydrocarbon chains	15,19
1455	δ CH_3 mainly from lipid hydrocarbon chains	15,19
1128, 1110	Carbohydrate and phosphate moieties	20
1065	νC-O, νC-C mainly from glyco(sphingo)lipids; P-O-C mainly of phospholipids	20-22,27
1044, 1032, 1030	νC-O, νC-C from carbohydrates / glyco(sphingo)lipids; P-O-C of phospholipids	20-22

Table S2. Assignment of the relevant IR components. In the Table, the peak positions from second derivative spectra have been reported for the spectral components identified by PLS-DA as relevant for the discrimination between tears from ALS patients and HCs . In addition, the main assignment to the tear biomolecules has been indicated.

REFERENCES

(1) Han, W.; Lin, Z. Learning from "Coffee Rings": Ordered Structures Enabled by Controlled Evaporative Self-Assembly. *Angew. Chem. Int. Ed.* **2012**, *51* (7), 1534–1546. https://doi.org/10.1002/anie.201104454.

(2) Filik, J.; Stone, N. Investigation into the Protein Composition of Human Tear Fluid Using Centrifugal Filters and Drop Coating Deposition Raman Spectroscopy. *J. Raman Spectrosc.* **2009**, *40* (2), 218–224. https://doi.org/10.1002/jrs.2113.

(3) Annarelli, C. C.; Fornazero, J.; Bert, J.; Colombani, J. Crack Patterns in Drying Protein Solution Drops. *Eur. Phys. J. E* **2001**, *5* (1), 599–603. https://doi.org/10.1007/s101890170043.

(4) Bassan, P.; Kohler, A.; Martens, H.; Lee, J.; Byrne, H. J.; Dumas, P.; Gazi, E.; Brown, M.; Clarke, N.; Gardner, P. Resonant Mie Scattering (RMieS) Correction of Infrared Spectra from Highly Scattering Biological Samples. *Analyst* **2010**, *135* (2), 268–277. https://doi.org/10.1039/B921056C.

(5) Pérez-Enciso, M.; Tenenhaus, M. Prediction of Clinical Outcome with Microarray Data: A Partial Least Squares Discriminant Analysis (PLS-DA) Approach. *Hum. Genet.* **2003**, *112* (5), 581–592. https://doi.org/10.1007/s00439-003-0921-9.

(6) Ripley, B. D. *Pattern Recognition and Neural Networks*; Cambridge University Press: Cambridge, 1996. https://doi.org/10.1017/CBO9780511812651.

(7) Ioffe, S.; Szegedy, C. Batch Normalization: Accelerating Deep Network Training by Reducing Internal Covariate Shift. In *Proceedings of the 32nd International Conference on Machine Learning*; Bach, F., Blei, D., Eds.; Proceedings of Machine Learning Research; PMLR: Lille, France, 2015; Vol. 37, pp 448–456.

(8) Friedman, J.; Hastie, T.; Tibshirani, R. Additive Logistic Regression: A Statistical View of Boosting (With Discussion and a Rejoinder by the Authors). *Ann. Stat.* **2000**, *28* (2), 337–407. https://doi.org/10.1214/aos/1016218223.

(9) Friedman, J. H. Greedy Function Approximation: A Gradient Boosting Machine. *Ann Stat.* **2001**, *29* (5), 1189–1232. https://doi.org/10.1214/aos/1013203451.

(10) Breiman, L.; Friedman, J.; Stone, C. J.; Olshen, R. A. *Classification and Regression Trees*; The Wadsworth and Brooks-Cole statistics-probability series; Taylor & Francis, 1984.

(11) Fawcett, T. An Introduction to ROC Analysis. *Pattern Recognit. Lett.* **2006**, *27* (8), 861–874. https://doi.org/10.1016/j.patrec.2005.10.010.

(12) de Oña, J.; Garrido, C. Extracting the Contribution of Independent Variables in Neural Network Models: A New Approach to Handle Instability. *Neural Comput. Appl.* **2014**, *25* (3), 859–869. https://doi.org/10.1007/s00521-014-1573-5.

(13) Louppe, G.; Wehenkel, L.; Sutera, A.; Geurts, P. Understanding Variable Importances in Forests of Randomized Trees. In *Proceedings of the 26th International Conference on Neural Information Processing Systems - Volume 1*; NIPS'13; Curran Associates Inc.: Red Hook, NY, USA, 2013; pp 431–439.

(14) Lê Cao, K.-A.; Boitard, S.; Besse, P. Sparse PLS Discriminant Analysis: Biologically Relevant Feature Selection and Graphical Displays for Multiclass Problems. *BMC Bioinformatics* **2011**, *12* (1), 253. https://doi.org/10.1186/1471-2105-12-253.

(15) Casal, H. L.; Mantsch, H. H. Polymorphic Phase Behaviour of Phospholipid Membranes Studied by Infrared Spectroscopy. *BBA - Rev. Biomembr.* **1984**, *779* (4), 381–401. https://doi.org/10.1016/0304-4157(84)90017-0.

(16) Barth, A. Infrared Spectroscopy of Proteins. *Biochim. Biophys. Acta-Bioenerg.* **2007**, *1767* (9), 1073–1101. https://doi.org/10.1016/j.bbabio.2007.06.004.

(17) Seshadri, S.; Khurana, R.; Fink, A. L. Fourier Transform Infrared Spectroscopy in Analysis of Protein Deposits. *Methods Enzymol.* **1999**, *309*, 559–576. https://doi.org/10.1016/S0076-6879(99)09038-2.

(18) Tamm, L. K.; Tatulian, S. A. Infrared Spectroscopy of Proteins and Peptides in Lipid Bilayers. *Q. Rev. Biophys.* **1997**, *30* (4), 365–429. https://doi.org/10.1017/S0033583597003375.

(19) Arrondo, J. L. R.; Goñi, F. M. Infrared Studies of Protein-Induced Perturbation of Lipids in Lipoproteins and Membranes. *Chem. Phys. Lipids* **1998**, *96* (1–2), 53–68.

https://doi.org/10.1016/s0009-3084(98)00080-2.
(20) Wiercigroch, E.; Szafraniec, E.; Czamara, K.; Pacia, M. Z.; Majzner, K.; Kochan, K.; Kaczor, A.; Baranska, M.; Malek, K. Raman and Infrared Spectroscopy of Carbohydrates: A Review. *Spectrochim. Acta. A. Mol. Biomol. Spectrosc.* **2017**, *185*, 317–335. https://doi.org/10.1016/j.saa.2017.05.045.
(21) Kirschbaum, C.; Greis, K.; Mucha, E.; Kain, L.; Deng, S.; Zappe, A.; Gewinner, S.; Schöllkopf, W.; von Helden, G.; Meijer, G.; Savage, P. B.; Marianski, M.; Teyton, L.; Pagel, K. Unravelling the Structural Complexity of Glycolipids with Cryogenic Infrared Spectroscopy. *Nat. Commun.* **2021**, *12* (1), 1201. https://doi.org/10.1038/s41467-021-21480-1.
(22) Wu, L. N. Y.; Genge, B. R.; Wuthier, R. E. Analysis and Molecular Modeling of the Formation, Structure, and Activity of the Phosphatidylserine-Calcium-Phosphate Complex Associated with Biomineralization. *J. Biol. Chem.* **2008**, *283* (7), 3827–3838. https://doi.org/10.1074/jbc.M707653200.
(23) Rygula, A.; Majzner, K.; Marzec, K. M.; Kaczor, A.; Pilarczyk, M.; Baranska, M. Raman Spectroscopy of Proteins: A Review. *J. Raman Spectrosc.* **2013**, *44* (8), 1061–1076. https://doi.org/10.1002/jrs.4335.
(24) Czamara, K.; Majzner, K.; Pacia, M. Z.; Kochan, K.; Kaczor, A.; Baranska, M. Raman Spectroscopy of Lipids: A Review. *J. Raman Spectrosc.* **2015**, *46* (1), 4–20. https://doi.org/10.1002/jrs.4607.
(25) Jacob Filik; Nicholas Stone. Raman Point Mapping of Tear Ferning Patterns; 2008; Vol. 6853. https://doi.org/10.1117/12.786441.
(26) Liu, J.; Conboy, J. C. Structure of a Gel Phase Lipid Bilayer Prepared by the Langmuir-Blodgett/Langmuir-Schaefer Method Characterized by Sum-Frequency Vibrational Spectroscopy. *Langmuir* **2005**, *21* (20), 9091–9097. https://doi.org/10.1021/la051500e.
(27) Kačuráková, M.; Mathlouthi, M. FTIR and Laser-Raman Spectra of Oligosaccharides in Water: Characterization of the Glycosidic Bond. *Carbohydr. Res.* **1996**, *284* (2), 145–157. https://doi.org/10.1016/0008-6215(95)00412-2.
